# Automated data mining of a plan‐check database and example application

**DOI:** 10.1002/acm2.12396

**Published:** 2018-06-29

**Authors:** Leon Dunn, David Jolly

**Affiliations:** ^1^ Icon Cancer Centre – The Valley Mulgrave, Melbourne Vic Australia; ^2^ Icon Cancer Centre – Richmond Richmond, Melbourne Vic Australia

**Keywords:** Mobius3D, plan‐check, statistical process control, treatment planning

## Abstract

**Purpose:**

The aim of this work was to present the development and example application of an automated data mining software platform that preforms bulk analysis of results and patient data passing through the 3D plan and delivery QA system, Mobius3D.

**Methods:**

Python, matlab, and Java were used to create an interface that reads JavaScript Object Notation (JSON) created for every approved Mobius3D pre‐treatment plan‐check. The aforementioned JSON files contain all the information for every pre‐treatment QA check performed by Mobius3D, including all 3D dose, CT, structure set information, as well as all plan information and patient demographics. Two Graphical User Interfaces (GUIs) were created, the first is called Mobius3D‐Database (M3D‐DB) and presents the check results in both filterable tabular and graphical form. These data are presented for all patients and includes mean dose differences, 90% coverage, 3D gamma pass rate percentages, treatment sites, machine, beam energy, Multi‐Leaf Collimator (MLC) mode, treatment planning system (TPS), plan names, approvers, dates and times. Group statistics and statistical process control levels are then calculated based on filter settings. The second GUI, called Mobius3D organ at risk (M3DOAR), analyzes dose‐volume histogram data for all patients and all Organs‐at‐Risk (OAR). The design of the software is such that all treatment parameters and treatment site information are able to be filtered and sorted with the results, plots, and statistics updated.

**Results:**

The M3D‐DB software can summarize and filter large numbers of plan‐checks from Mobius3D. The M3DOAR software is also able to analyze large amounts of dose‐volume data for patient groups which may prove useful in clinical trials, where OAR doses for large numbers of patients can be compared and correlated. Target DVHs can also be analyzed *en* mass.

**Conclusions:**

This work demonstrates a method to extract the large amount of treatment data for every patient that is stored by Mobius3D but not easily accessible. With scripting, it is possible to mine this data for research and clinical trials as well as patient and TPS QA.

## 1 INTRODUCTION

Recent reports have estimated that the U.S. healthcare data accumulation reached 150 exabytes (10^18^ bytes) in 2011, with growth models predicting this to exceed a zettabyte (10^21^) and eventually a yottabyte (10^24^) of data every year.^1^ To put this in context, the capacity of the human brain has been estimated at 1 petabyte (10^15^)^2^ and thus to store this accumulation of data, 1 billion individuals would be required. Historically, the utilization of this data has been low but the future potential is well established.^1^ In the radiation oncology health space, the use of database systems has been reported for long (25 years)^3^ and short (5 years)^4^ periods, as well as for specific treatment sites.^5^ On a global level, the reported advantages ranged from informing treatment policy, resource allocation, clinical trials assessment, enhancing research and the publication of peer‐reviewed papers.^3^, ^4^, ^5^ For optimal outcomes, these publications also allude to the need for these database tools to be automated and streamlined in the collection, analysis, and presentation of large amounts of data. The majority of this data is currently stored within the Oncology Information System (OIS) and commercial database analytics services such as InSightive (Varian Medical Systems) are available. These solutions can often be limited in the ability to customize analytic solutions, especially when it comes to advanced statistical analysis.

Mobius3D is commercially available independent dose verification software from Mobius Medical Systems (Mobius Medical Systems, LP, Houston, TX, USA). Unlike previous point dose verification software packages, Mobius3D utilizes the full patient DICOM set (CT, plan, structures, and dose) to recalculate dose in three dimensions using a collapsed cone convolution superposition^6^ and independent reference beam data. The software presents results as dose‐volume histogram (DVH) comparisons for regions‐of‐interest (ROIs), target coverage (mean dose, 90% coverage), and 3D gamma comparisons for the dose‐calculation volume comparing both treatment planning system (TPS) and Mobius3D's secondary dose calculation. The commissioning and use of Mobius3D has been reported elsewhere.^7^, ^8^, ^9^, ^10^, ^11^, ^12^, ^13^

By including the patient anatomy and having independent beam data and dose calculation algorithm, Mobius3D not only provides a robust second check of the treatment plan prior to the patient being treated, but can also inform clinical decision making due to more subtle TPS limitations and uncertainties.^14^ One aspect of Mobius3D, which to the author's knowledge has perhaps not yet been explored, is the fact that all of the patient's plan data used to calculate the result and present the report is also stored by Mobius3D.

CT data, structure sets, plan parameters (field sizes, gantry angles, couch angles, beam energy, MUs, MLC data), patient demographics, dose‐volume, and fractionation information are all recorded. While almost all of these data are also stored in the OIS, it can be difficult to access at a database level and does not offer the additional benefit of also having independent verification data. As a result, there is a vast amount of data those originate in the TPS and is then stored within the Mobius3D database, but is not necessarily scrutinized on a global level.

In the preface to this work, results obtained during commissioning of the system have been presented.^14^ In that work, results from the first 1000 patients were used to modify the tolerance values relating to pass and action limits for plan‐check results depending on the calculation algorithm (superposition‐convolution or pencil beam) and treatment site. This initial work was primarily based on manual spreadsheet input. The work presented herein builds on this through scripting and GUIs that are used to automate the retrieval, analysis, and presentation of any data of interest. As an example application for this automated data retrieval, statistical process control (SPC)^15^, ^16^, ^17^ methods can be employed on the comparison between Mobius3D and the TPS with respect to the mean dose to the PTV.

At our institution, every patient's approved treatment plan is sent to Mobius3D prior to treatment regardless of the complexity of technique. The Mobius3D report is then either checked by a radiation therapist for 3D conformal radiation therapy or by a Physicist for dynamic or stereotactic treatments. The Mobius3D check is approved and a report is generated and stored with the patient's file in the OIS.

As every patient's final treatment plan transits through the Mobius server and all of the plan information is retained, there is automatically a large amount of untapped data which can be accessed through scripting. The logical next step is to utilize this data to obtain global plan information for patient populations, dose distribution and OAR statistics, or even patient throughput analyses for facilities management.

The aim of this work was to present this software and its current capabilities. As of April 2017, 4091 approved Mobius3D checks have been completed, providing a large dataset to explore. The authors plan to make this software available and see the potential future use of this software for large scale plan credentialing in the clinical trials setting, clinical trial arm comparisons (where OARs are of interest), correlation studies, meta‐analysis, TPS QA, and even remote auditing of radiotherapy center TPSs.^18^, ^19^, ^20^

## 2 MATERIALS AND METHODS

### 2.A Mobius3D and data storage

Mobius3D runs on stand‐alone server architecture with graphics‐processing unit (GPU) capabilities and provides a secondary dose calculation using the same CT data, structure set and RT plan used by the TPS. The value of this secondary dose calculation is that it is fully 3D and uses a proprietary dose calculation algorithm whose input is consensus beam data, rather than institution specific beam data collected by the user, as used by the TPS. Mobius3D compares its own dose calculation result to that of the TPS and presents a comprehensive report encompassing dose‐volume metrics, gamma, and coverage statistics. The end‐user can specify warning and out‐of‐tolerance levels for mean dose and 90% coverage as well as the percentage 3D gamma pass‐rate. DVH constraints are taken from the literature (RTOG publications, AAPM TG 101) and are fractionation dependent (conventional vs stereotactic).

Mobius3D stores all plan data needed for calculation and presentation of results in the form of a JavaScript Object Notation (JSON) file. This data format is an open‐standard, human readable text consisting of attribute‐value pairs and data array types similar to XML. Table 1 shows the basic data structure and major fields that are stored for each patient. Note that for each patient two .JSON files are stored on the server, a .JSON file containing all of the plan data and a .JSON file containing all of the dose‐volume data for all structures listed in the plan that has been previously assigned in Mobius3D.

**Table 1:** Parameters stored for each patient that can be accessed for different patient groups along with full dose‐volume information

PLAN DATA .JSON File					DVH .JSON
Sub section: “Data”		“Request”	“Settings”		n/a
*Beam Plan Information*	*Results Information*	*Parameter*	*Mobius3D Settings*	*Patient Data*	*DVH data*
Segments	Target coverage	Approvals	Dose search window	Patient's sex	Structures
Machine	Mean dose	Institution	Coverage	Birth date	Mobius3D DVH
Delivery Time	3D gamma	Dates	Gamma	Patient ID	TPS DVH
Patient setup technique	ROI Names	Server diagnostics	Dose	Equipment (linac)	
Patient setup positions	ROI Dose	Plan name	Density override	Planner's name	
Control points	ROI Coverage	Timestamps	MU	Study description	
MLC positions	Gamma ROI	Notes	MLC	Study date	
MLC type	Slice dose information		Curative/Palliative intent	Physician	
Jaws	OAR constraints		Jaw		
Energy	Mean dose per ROI		Couch		
Deliverability	No. of fractions		Collimator		
Collimator angles			CBCT		
Gantry angles			Gamma cut‐off		
Applicator					
Beam name					

### 2.B. Python and matlab

Python is used to query the server and copy plan data .JSON files and DVH .JSON files to a disk location. Note: This is an extraneous step and could be done through matlab (MathWorks^®^, Natick, MA, USA) if it was running on the internal network. In this work, matlab was used to create two separate graphical user interfaces (GUIs), Mobius3D‐Database (M3D‐DB) and Mobius3D organ at risk (M3DOAR), as well as process and analyze the .JSON files stored by Mobius3D.

### 2.C. GUI—Mobius3D Database “M3D‐DB”

**Figure 1:**
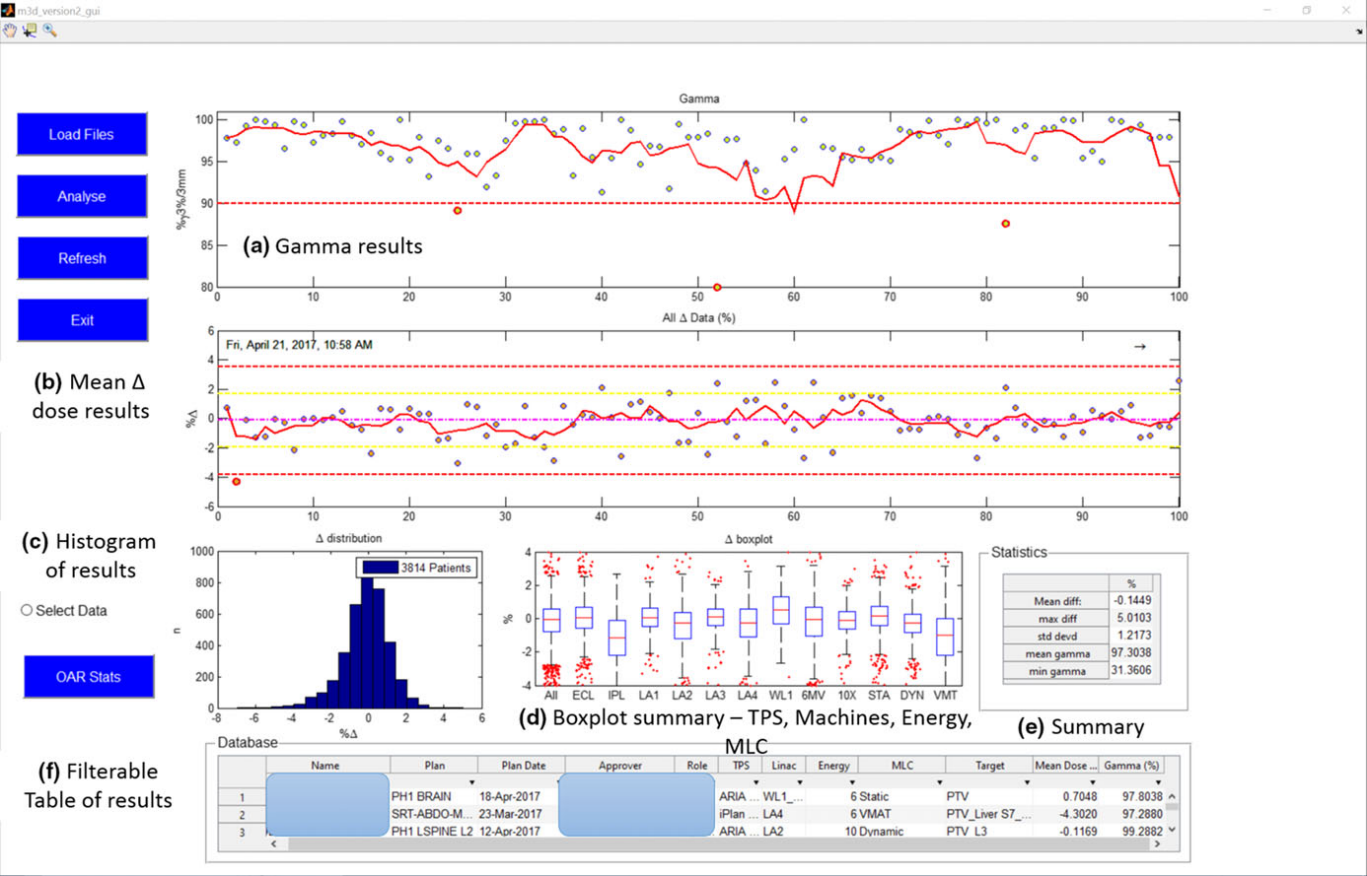
The M3D‐DB software GUI showing a timeline of gamma results (a). (b) Mean dose discrepancies between Mobius3D and the TPS. (c) A histogram of mean dose discrepancies. (d) Additional boxplots of all plan‐check results (mean dose discrepancy) are shown for both planning systems (ECL—Eclipse, IPL—iPlan), individual linacs (LA1, 2, 3, 4, WL1) and all beam energies (6MV, 10MV) and MLC characteristics (static, dynamic, VMAT). Summary statistics and the filterable table of results are shown in (e) and (f), respectively. Names of patients and approvers have been blocked out for confidentiality.

The M3D‐DB GUI is shown in Fig. 1. Once the group is analyzed by selecting the *Analyze* button, the GUI presents the data in the form of time series of both the 3D gamma results and differences in the mean dose to the PTV as reported by Mobius3D and the TPS, respectively. A Microsoft Excel spreadsheet is also created as a secondary backup of data. A histogram of the mean dose difference distribution for all patients processed as well as a boxplot summary of all results (mean dose difference) and summary statistics is also presented. The summary statistics box includes mean, maximum and standard deviation differences between Mobius3D and the TPS for the PTV mean dose over the entire (or filtered) cohort of plan‐checks. Gamma results are summarized by the mean and minimum gamma pass‐rates for the selected plan‐check cohort.

The gamma and mean dose discrepancy results are interactively scrollable, zoomable, and selectable. By toggling the *Select Data* radio button, a crosshair is presented and the user can select any data point. Clicking on the point opens a web browser and the patient's plan‐check in Mobius3D. This feature is particularly useful for out of tolerance results where the user can review the plan‐check in more detail via Mobius3D and discern the reasons for potential out‐of‐tolerance results.

Filtering and sorting the data to display is achieved by the use of drop down boxes or wildcard search queries to interrogate the table of results. Selecting the *Refresh* button will then update the data presented. Using wildcard search options allows for inconsistency in nomenclature used by staff when naming plans. For example, typing *PROS into the Plan drop down field will search the table for any plan names that contain the string “PROS” in lower or upper‐case. This will yield all PROS, PROSTATE, PH1_prostate, PH1 Pros etc.

For each patient plan check the following information is recorded in separate variables, which are displayed and added to the Excel database.

- Patient Name
- Plan Name
- Approval date
- Approver
- Role
- Treatment planning system
- Treatment machine name
- Beam Energy
- MLC (static, dynamic, VMAT)
- Treatment target name (PTV etc.)
- Mean Dose difference to the target
- Gamma Pass rate

To not skew the SPC analysis, if there are multiple targets (gross, clinical and planning target volumes) only one target per patient is considered. For multiple target volumes (as is the case for the majority of patients), the primary target is defined as the one receiving the highest mean dose and the analytics are only recorded for this volume. SPC limits are applied to the processed mean dose (*μ*) difference data with default values of mean plus and minus three standard deviations (*μ *± 3*σ*) for out‐of‐tolerance and ±2*σ* for the warning level, respectively. These limits are customizable and adapt to the data of interest based on the table filter values, however the application of SPC to secondary dosimetry checks is not the aim of this paper and limit specification/sampling parameters are to be explored fully in future work.

### 2.D. GUI—Mobius3D organ at risk “M3DOAR”

**Figure 2:**
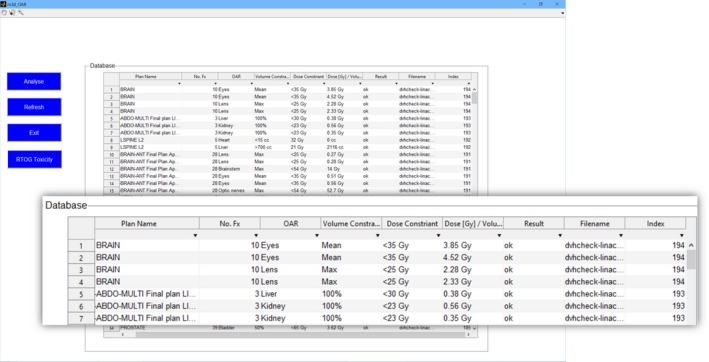
M3DOAR software GUI. A table of plan names, number of fractions, OAR name, volume constraint, dose constraint, dose/volume value, result, filename ID, and an index value is presented. An enlarged view of the database itself is shown inset.

The M3DOAR GUI is shown in Fig. 2. This software analyses the DVH .JSON files produced by every plan‐check. These .JSON files are read and passed by the M3D‐DB software while it is reading in the plan‐check .JSON file associated with a particular patient. A script then collects the relevant parameters relating to plan names, number of fractions, volume, and dose constraints, and results. The script also retrieves the names of the OARs which Mobius3D has automatically assigned as ROIs to be included in the generated Mobius3D report.

For each DVH .JSON file, the complete dose‐volume information for all plan structures calculated with both Mobius3D and the TPS is recorded and accessible. The software works by extracting and cataloguing the Mobius3D results for each ROI/OAR for every patient loaded and then allowing searching and filtering through tables to present the DVH data and statistics relevant to the user's selection.

The user can filter the data based on the plan name, fractionation, dose constraint, etc. and then use search term or drop‐down selection to scan and retrieve particular OAR dose‐volume data. Search strings allow users to search for the original TPS designated names which Mobius stores, but does not present. Again, these are necessary to cope with inconsistent nomenclature for contoured structures.

As an example, typing “Rectum” into the search field and clicking Refresh will scan each patient's DVH .JSON file in the current (filtered/not filtered) list and plot the associated DVH data. The software prompts the user to graphically select a particular dose and volume constraint and an RTOG toxicity button brings up reference toxicity data employed by Mobius3D for its out‐of‐tolerance limits. Once the DVH constraints are selected, every plan‐check that contains a “Rectum” OAR will have the DVH data and histograms of the minimum absorbed dose at volume *V*,* D(V)*, percentage volume that received dose *D, V(D)*, and the distribution of maximum doses to the structure plotted. The mean, median, and (*μ*±1*σ*) are calculated and also plotted.

### 2.E. Clinical workflow

**Figure 3:**
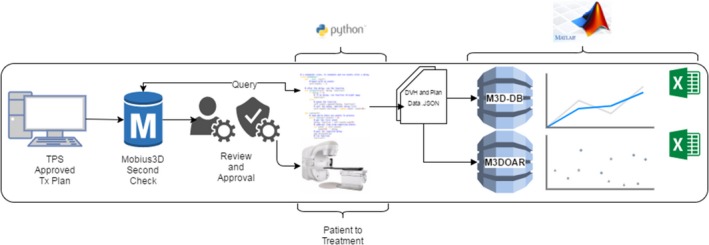
Schematic of the workflow in use at our institution.

The basic workflow for the use of Mobius3D at our facility is shown in Fig. 3. All approved patient plans have their CT, RT structure set and RT dose exported from the TPS (Varian Eclipse, Brainlab iPlan) to Mobius3D. Mobius3D then performs a full‐3D dose calculation and compares the result to that of the TPS. At this stage, either a Radiation Therapist or Medical Physicist checks the report and approves or investigates warning and out‐of‐tolerance results. If the plan is approved, the patient proceeds to treatment. At any point in time, following the approval of the Mobius3D check, the database can be queried using a python script to retrieve the last *n* approved plan‐checks in the background. The presence of an approval stamp assures that only final, approved plan reports are retrieved for inclusion in the database and any resulting analysis. The aforementioned GUIs then run in matlab and are used to read in and process the .JSON files obtained from the Mobius3D server.

### 2.F. Data validation

Data validation was carried out by manually validating each plan‐check data point downloaded to the GUI for a subset of the total dataset. Each point was chosen at random from the time‐series of results using the interactive *Select Data* button which allows users to select a data‐point and be taken to the identified plan on the Mobius3D server for comparison. Each parameter in the M3D‐DB and M3DOAR data‐tables was then compared against the actual value in Mobius3D. No incorrect linkages, timestamp transfer or loss of precision was observed. All data analysis and presentation was performed using existing functions in matlab.

## 3 RESULTS

### 3.A M3D‐DB: analysis of plan‐check data

**Figure 4:**
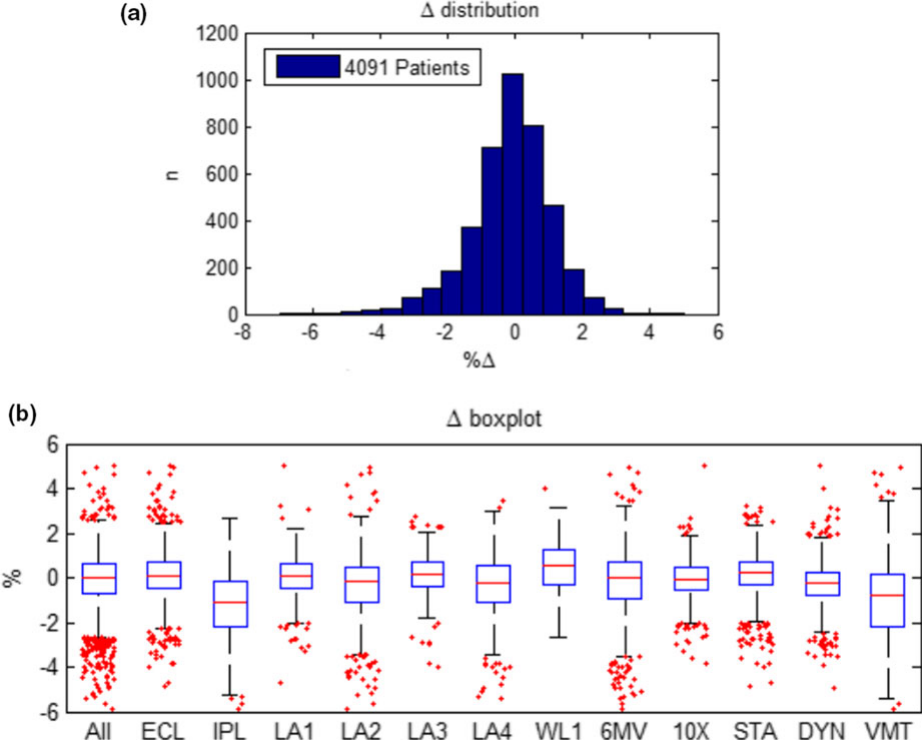
(a) A histogram showing the distribution of mean dose differences between Mobius3D and the TPS for 4091 approved plan‐checks since April 2014. (b) A boxplot of the mean dose discrepancy results for all the combinations at our facility. 2x planning systems, 5x linacs with 2x photon energies and 3x MLC modes are shown. ALL = All data, ECL = Eclipse, IPL = iPlan, LA1, 2, 3 and 4 =  Linear Accelerators 1–4, 6MV = photon energy, 10X = photon energy, STA = Static MLCs, DYN = Dynamic MLCs, VMT = volumetric modulated arc therapy. On each boxplot, the central mark indicates the median, the bottom and top edges indicate the 25th and 75th percentiles, respectively. Whiskers extend to the maximum and minimum values and outliers are plotted individually using the “+” symbol.

Fig. 4(a) shows the results of 4091 plan‐checks with Mobius3D presented as a histogram. This histogram is comprised of unfiltered data and therefore contains two planning systems, five linacs with two photon energies each and static, dynamic, and VMAT MLC modes. The average difference between the mean doses to the PTV as calculated by the TPS and M3D for all plan checks was found to be −0.13% ± 1.2% (1*σ*). The maximum difference for all plan‐checks to date was 5.0% and the mean gamma pass‐rate (3%/3 mm) was 98%.

Fig. 4(b) shows this data separated into boxplots as presented in M3D‐DB. It can be seen that depending on the planning system, machine, energy, and MLC type the distribution of plan‐check results varies. This is to be expected as each planning system uses a different dose‐calculation algorithm and at our institution we have multiple linac models with varying MLC types. Note that this does not represent the total number of patients treated, as some patients are planned across multiple linacs.

### 3.B Example of use: tightening limits based on historical data

**Figure 5:**
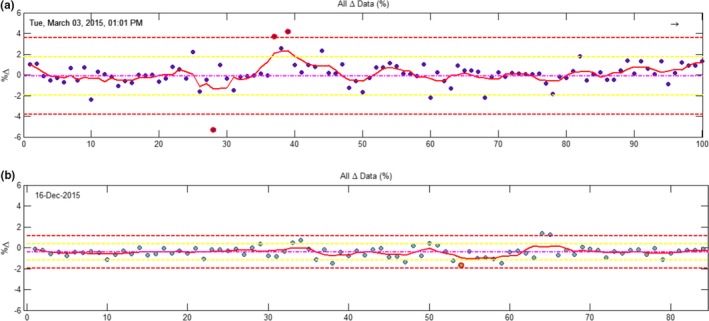
An example of setting the Mobius3D tolerances for warning (yellow) and out‐of‐tolerance (red) for the agreement in mean dose to the PTV based on all historical results (a) and (b) tailoring the limits based on the treatment site, in this case, for all prostate patients.

**Figure 6:**
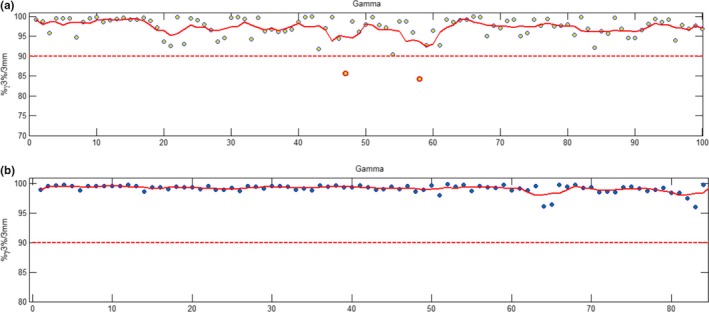
3D gamma result (3%/3 mm) plan‐checks analyzing all data (a) versus gamma results for prostate plans only (b). It is clear that the prostate warning and action levels can be tightened even based on ~ 100 patients. Note: In the GUIs these charts are scrollable with the x‐axes limits being clipped here for presentation purposes. Here, a hard‐coded limit of 90% out of tolerance limit is shown.

Figs. 5 and 6 show examples of tightening warning and out‐of‐tolerance values based on the particular treatment site. This can also be expanded to machine, planning system, energy, and any other parameter, although, Mobius3D does not currently support this level of customization and therefore limits have to be implemented in‐ house. Fig. 5(a) shows data discrepancies in mean dose to the PTV as calculated with the TPS and Mobius3D for all treatment sites, planning systems, linacs etc. Fig. 5(b) shows warning and out‐of‐tolerance limits as calculated for prostate plans only. It can be seen that by tailoring the tolerance limits to particular treatment sites, the warning, and out‐of‐tolerance limits are reduced by approximately half. By tightening these limits, there is potential to detect more subtle planning variations and system limitations with Mobius3D, rather than just gross errors prior to treatment. Fig. 6(a) and (b) show 3D Gamma results for (a) all patients to date (*n *=* *4091) unfiltered and (b) results for prostate patients only.

### 3.C M3DOAR: analysis of DVH data

**Figure 7:**
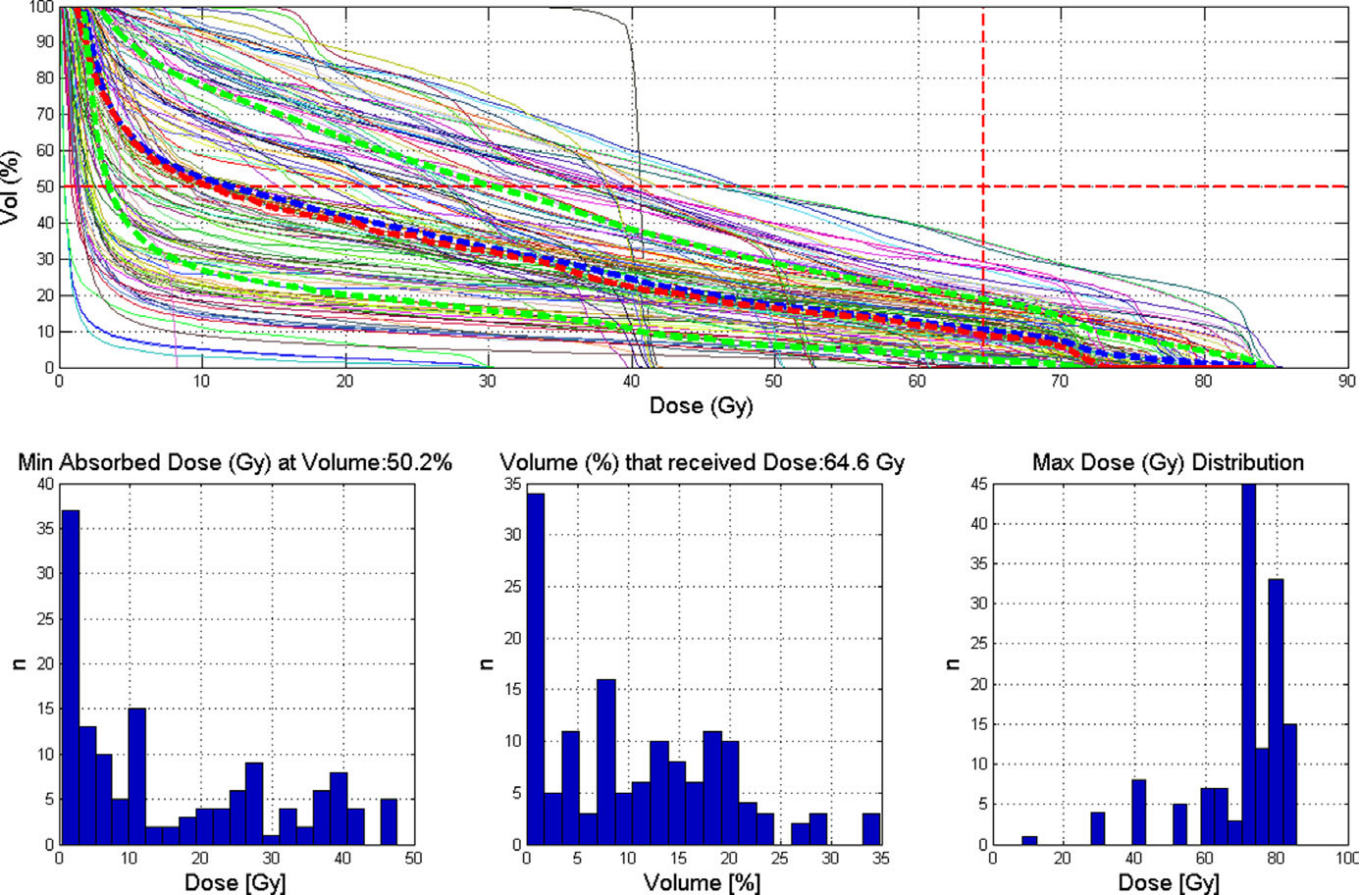
Example of filtering out TPS DVH data for the bladder volume for prostate patients. Shown here is the TPS DVH data for every patient found, along with the mean, median and *μ* ±1*σ*, shown as thick blue dashed lines, thick red dashed line and thick green dashed lines, respectively. Also shown here are histograms based on the selected dose‐volume constraints (dashed crosshair) of approximately 65 Gy and 50%, respectively.

**Figure 8:**
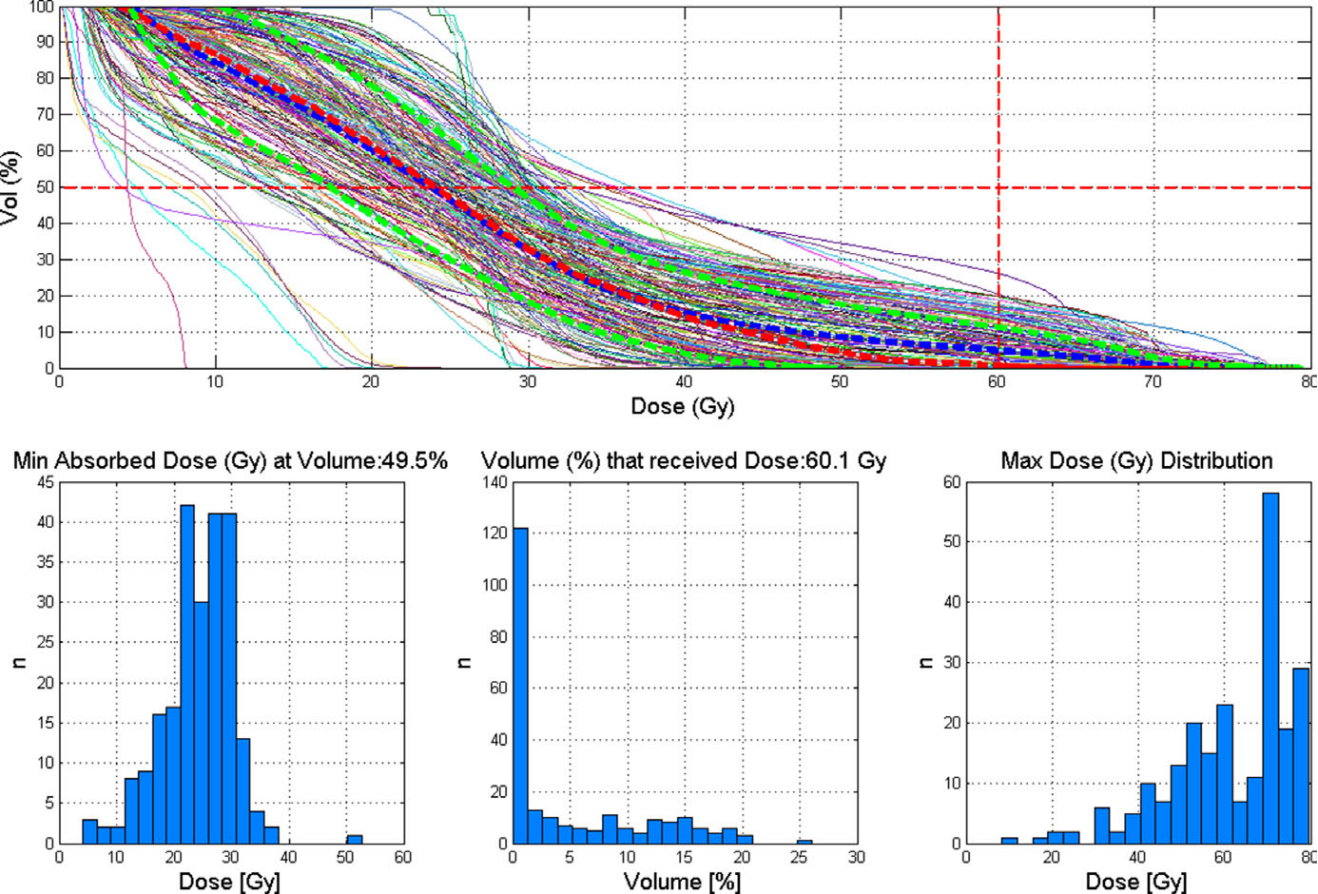
Another example of filtering out TPS DVH data, this time for the rectum volume for prostate patients. Shown here is the TPS DVH data for every patient found, along with the mean, median, and *μ* ±1*σ*, shown as thick blue‐dashed lines, thick red dashed line and thick green dashed lines, respectively. Also shown here are histograms based on the selected dose‐volume constraints (dashed crosshair) of approximately 60 Gy and 50%, respectively.

Two examples of the use of the M3DOAR software are shown below in Figs. 7 and 8. These examples highlight the type of meta‐analyses that can be performed with M3DOAR. Here, bladder and rectum TPS DVHs are shown with customizable dose and volume constraints selected by the user. Additional histograms showing the distributions of doses and volumes are also displayed based on the selected constraints.

Here, bladder and rectum data (*n *=* *140) for prostate patients are presented in DVH and histogram form with histograms based on the user‐chosen dose and volume constraints shown as dashed horizontal and vertical lines on the DVH plot. The following histograms are presented:

1. The distribution of minimum doses (Gy) at user‐selected volume (%)
2. The distribution of volume (%) that received user‐selected dose (Gy)
3. The distribution of maximum doses (Gy)

## 4 DISCUSSION

Advances in computing power, database storage methods, statistical investigation, and the ability for multiple, independent centers to share data has allowed for the creation of large data‐sets, termed “big data” to become a possibility in the radiotherapy and oncologic space. Patient demographics, diagnoses, genetic, imaging, treatment, and outcome information are all ascertained in the process of a patient's therapy. It is how this data is stored, sorted, analyzed, and interpreted in bulk that is a driving research question that will no doubt have an impact on clinical research, treatment methods, and treatment outcomes.^21^

While there is no doubt enthusiasm about the possibility of transformative outcomes in this setting, the “big data” era faces a number of challenges. Accumulating large and diverse enough clinical data‐sets required to predict outcomes, influence clinical decisions, and create patient population prediction models needs collaboration between organizations.^22^ This task in itself poses a logistical and administrative challenge, before even progressing to the stage of analyzing and interpreting the data itself. In addition to this, ethical and legal considerations as well as privacy concerns are all problems that require careful consideration in the area of “big data”, all of which have been discussed in the literature.^1^, ^21^, ^23^

Sivarajah *et al* 24 separates the challenges facing big data into three categories:

1. Data challenges—volume, variety, quality, volatility
2. Process challenges—capturing, integrating, analyzing, feedback, modeling and prediction
3. Management challenges—privacy, security, governance, ethics and legal considerations

In this technical note, we aimed to present and demonstrate a software tool that allows meta‐analyses of plan‐check and dose‐volume data created through the Mobius3D secondary dose calculation software. Although this software manages, stores, analyses, and interprets data, it is only doing so for one aspect of the available data that a single patient's treatment necessitates. Nonetheless, the challenges presented by Sivarajah, particularly points one and two, were faced during development and required careful consideration and technical software engineering to overcome.

With regard to the potential applications of this type of software, both M3D‐DB and M3DOAR could find valuable potential use in clinical trials for ensuring plan quality and evaluating dose‐volume data. Patient sub‐groups within clinical trials can also be analyzed and compared with the software. Further to this, there is scope to develop the tool as a remote auditing platform. An auditing service^19^, ^25^, ^26^, ^27^, ^28^, ^29^ could run Mobius3D and allow facilities to send a specifically created plan prepared on a phantom to the server. As each facility TPS is configured with in‐house collected beam data, it is possible to independently evaluate the dosimetric consistency between centers remotely, using the independent consensus beam data employed by Mobius3D.

The software could also be used to provide a method for automated and continuous QA of TPSs. The hypothesis for this use is that if variable parameters that drive the TPS dose‐calculation are incorrect or inadvertently modified, an analysis of sequential plan‐checks using M3D‐DB should yield an out of control process that can then be investigated further.

Automation is important for maximizing the benefit of a database while minimizing the labor required maintaining and interrogating it. At the time of publication, this software is setup independently from the Mobius3D server. However, a version is being created that will run on the network and automatically query the Mobius3D database once a new plan‐check is completed. The role of the software will then be a quality assurance tool, comparing the current plan‐check against all previous plan‐checks for identical parameters using SPC and alerting the Physics personnel to investigate outliers, all the while collating and collecting dose‐volume and patient plan information. This automation could also extend to DVH data comparing a DVH for an OAR to all existing DVH data for that organ.

## 5 CONCLUSION

This technical note presents the development and use of software that allows for meta‐analyses of plan‐check and DVH data obtained and processed by Mobius3D. The scope of this work in the era of “big data” in healthcare is niche and addresses only a portion of the data available for analyses, per patient throughout a course of radiotherapy. However, the potential of such a database and analysis framework to be automated provides an additional layer of quality control at no additional cost in labor. The database generated expands in size with the natural progression of each patient to their treatment and could potentially feed into larger clinical databases for imaging, demographic, diagnoses, and outcome cross‐correlation.

## CONFLICT OF INTEREST

No funding has been received from Mobius Medical Systems relating to the development of this software and this publication. No financial arrangement is in place for potential future financial benefits relating to this software.
